# Kinomics toolbox—A web platform for analysis and viewing of kinomic peptide array data

**DOI:** 10.1371/journal.pone.0202139

**Published:** 2018-08-21

**Authors:** Alex M. Dussaq, Timothy Kennell, Nicholas J. Eustace, Joshua C. Anderson, Jonas S. Almeida, Christopher D. Willey

**Affiliations:** 1 Department of Radiation Oncology, The University of Alabama at Birmingham, Birmingham, Alabama, United States of America; 2 Informatics Institute, Department of Medicine, The University of Alabama at Birmingham, Birmingham, Alabama, United States of America; 3 Department of Biomedical Informatics, Stony Brook University School of Medicine, Stony Brook, New York, United States of America; International Centre for Genetic Engineering and Biotechnology, INDIA

## Abstract

Kinomics is an emerging field of science that involves the study of global kinase activity. As kinases are essential players in virtually all cellular activities, kinomic testing can directly examine protein function, distinguishing kinomics from more remote, upstream components of the central dogma, such as genomics and transcriptomics. While there exist several different approaches for kinomic research, peptide microarrays are the most widely used and involve kinase activity assessment through measurement of phosphorylation of peptide substrates on the array. Unfortunately, bioinformatic tools for analyzing kinomic data are quite limited necessitating the development of accessible open access software in order to facilitate standardization and dissemination of kinomic data for scientific use. Here, we examine and present tools for data analysis for the popular PamChip^®^ (PamGene International) kinomic peptide microarray. As a result, we propose (1) a procedural optimization of kinetic curve data capture, (2) new methods for background normalization, (3) guidelines for the detection of outliers during parameterization, and (4) a standardized data model to store array data at various analytical points. In order to utilize the new data model, we developed a series of tools to implement the new methods and to visualize the various data models. In the interest of accessibility, we developed this new toolbox as a series of JavaScript procedures that can be utilized as either server side resources (easily packaged as web services) or as client side scripts (web applications running in the browser). The aggregation of these tools within a Kinomics Toolbox provides an extensible web based analytic platform that researchers can engage directly and web programmers can extend. As a proof of concept, we developed three analytical tools, a technical reproducibility visualizer, an ANOVA based detector of differentially phosphorylated peptides, and a heatmap display with hierarchical clustering.

## Introduction

Kinases are fundamental to cellular life; they provide essential regulation and function in nearly every pathway. Due to this, there has been increasing interest in investigating kinases on a global scale. Kinase based investigations generally focus on one of two major categories; (1) the phosphoproteome[1–4], the set of kinase targets and, (2) the kinome[5–9], the set of cellular kinases. Kinome analysis can focus on either quantification of kinase abundance or activity. Arguably, the most potential clinical relevance is in the measurement of kinase activity[6,7]. In general, kinome activity has been measured utilizing either mass spectrometry (MS) or peptide array methods. Due to the low abundance of kinases, MS requires enrichment or purification of kinases or their products to produce useful information. A variety of isolation techniques are used in conjunction with tandem MS to quantify and accurately measure kinase activity[7]. While these MS techniques are continuing to develop and gaining popularity, peptide arrays remain the more commonly used kinomic approach.

Covered thoroughly in the recent Baharani et al. review[9], three core peptide array technologies exist for measuring kinase activities. All three technologies affix phosphorylatable peptide residues to a different matrix; (1) glass beads in solution, (2) glass slide based, 2D kinome microarrays, and (3) porous 3D microarrays, PamGene PamChip^®^ [9]. The more developed PamChip and 2D kinome microarrays provide a high-throughput measure of kinase activity by exposing kinases to phosphorylatable peptide residues and utilizing either fluorescent antibodies or radioactive isotopes to detect phosphorylation amounts[6,9].

Our lab focuses on the PamChip system, that has arrays with at least 144 distinct peptides, each composed of 12–15 amino acids, with one or more phosphorylatable residues. The arrays are specifically designed to capture kinase assay data for either the tyrosine kinome (protein tyrosine kinase or PTK PamChip) or serine/threonine kinome (serine/threonine kinase or STK PamChip) with target peptides containing phosphorylatable tyrosine or serine/threonine residues, respectively. Peptides are printed onto the array in ‘spots’, and similar to other microarray technologies, phosphorylation is quantified utilizing image pixel brightness at each spot. To capture kinetic data, images are taken at set intervals over the course of a reaction. To capture end-level (post-wash) data, images are taken at varying camera exposure times. Each image is analyzed utilizing proprietary software that can report a number of parameters including median signal and background. Depending on the study, either median signal[10] or a median signal, background value[11–13] is then utilized for subsequent analyses.

While the PamChip system lacks the quantity of phosphorylatable peptide targets found on some complementary 2D kinome microarrays, it does provide unique data in the form of kinetic phosphorylation measurements during the multiplexed *in vitro* kinase assay. Unfortunately, the majority of published PamChip studies do not report kinetic data, but instead, describe only the end level data (e.g., end of reaction data points) albeit with upstream kinase prediction analyses[11,12,14]. We believe the underutilization of the kinetic data is due to its relative complexity[15,16]. Studies that do utilize the kinetic curves focus on the early slope (so-called initial velocity or V_ini_) and often do so in combination with another unique technique available, *ex vivo* addition of kinase inhibitors to cell lysate immediately prior, or during the assay. Kinase inhibitor inclusion allows for "biological interrogation" of the lysate by visualizing the reduction in specific phosphopeptide(s) intensities [11,17]. Nevertheless, kinetic curve data has largely been ignored in the literature.

Another major challenge facing the field is that of data management. A multitude of recent studies have identified opaque data analysis tools as a major problem [18]. Initiatives such as FAIR are now setting the ground rules for data and analytical products to be “Findable, Assessable, Interoperable, and Reproducible [19]. Unfortunately, development of FAIR analytic platforms for kinomics data is quite limited. Initial approaches for handling 2D kinome microarray data included two versions of an analysis platform, PIIKA, specifically designed for the PepScan system [20,21]. This platform was created utilizing R and Perl allowing users to upload annotated files and receive an analysis by email. BioNavigator [22], a licensed software package, can perform similar, but less transparent, analyses for PamChip data but requires domain expertise and manual interaction.

A related challenge in the field is the lack of kinomic data standardization as highlighted in two recent reviews [9,23]. Recommendations include adoption of a specialized version of Minimum Information About a Microarray Experiment (MIAME) standard[24] for kinomic publications, and a kinome specialty meeting to discuss standards, respectively[9,23]. A follow-up critique of MIAME indicates early shortfalls of this standard were the lack of simple data format standards and a lack of publicly available data sets[25].

Therefore, we have addressed these two major challenges by: 1) creating a series of web-based analysis and display tools for PamChip data in an open source format with transparent data handling for background normalization, curve fitting, data parameterization and outlier detection; and 2) by supplying a simple, but rigorous, JavaScript Object Notation (JSON) document-based data standard and instructions for creation of a MongoDB database. The “Kinome Toolbox” functionality is demonstrated through a case study of genetically modified glioblastoma (GBM) cells, a disease well known for aberrant kinase signaling.

## Methods

### Cell culture system

We have previously published on the expression and function of the protein Myristoylated Alanine Rich C-Kinase Substrate (MARCKS) in the human GBM cell line U87 including the generation and characterization of doxycycline-inducible MARCKS mutant U87 cell lines [26,27]. The specific alterations to this protein are shown schematically in S1 Fig, and are as follows:

1. Vector Control (CT): U87 transduced with an empty vector.
2. MARCKS overexpression (WT+): U87 transduced with the wild-type MARCKS protein which contains four phosphorylatable serine residues in its effector domain (ED).
3. Non-Phospho MARCKS (NP): U87 transduced with MARCKS whose phosphorylatable serine residues within the ED are mutated to non-phosphorylatable alanine's, thus mimicking the unphosphorylated state of MARCKS[27].
4. Pseudo-Phospho MARCKS (PP): U87 transduced with MARCKS whose phosphorylatable serine residues in the ED are mutated to aspartic acid, permanently mimicking the charge of phosphorylated serine residues[27].

The culture conditions have been previously described but briefly, 5 x 10^5^ of each mutant cell line were plated in biological triplicate and induced overnight with 1 μg/mL doxycycline in standard growth medium—Dulbecco’s modified Eagle’s medium (DMEM) supplemented with 10% fetal bovine serum (FBS). All cells were maintained at 37°C in 5% CO_2_.

### Kinomic assay

Cells were lysed according to standard operating procedures of The University of Alabama at Birmingham’s (UAB) Kinome Core similar to prior reports[11,28], with slight modification in order to assess the effects of measurement accuracy, background normalization, and saturation as described below. Samples were quantified for protein content and normalized to equivalent DNA levels and run in biological triplicate on the PamStation^®^12 housed in the UAB Kinome Core. There are two phases to the kinomic assay, the kinetic and end-level (post-wash) phases. The kinetic phase is indexed by cycles of sample fluid pumping with measurements beginning at cycle 32. The most commonly used method involves substrate phosphorylation image capture during every 4 subsequent cycles (for a total 92 cycles) at a single 50ms exposure time. Following this, the chip is washed of active lysate and 5 pictures are taken at varying exposure times (10ms, 20ms, 50ms, 100ms, 200ms) and labeled as post-wash data. For the case study presented here, we extended the chip run time to 152 cycles, taking pictures at 5 exposure times (10ms, 20ms, 50ms, 100ms, 200ms) every 6 cycles during the kinetic data portion, then captured the traditional 5 post-wash images. All images were then processed by the Evolve2 (0.08)[22] software to extract separate median signal and median background measurements. This data was then exported with all metadata as 2 ‘BioNavigator crosstab’ files for subsequent Kinome Toolbox as detailed below.

### General data workflow

The proposed workflow for preparation for data analysis is briefly described here and shown schematically in S2 Fig. All steps are described thoroughly in other sections and unless otherwise indicated can be performed stepwise using client-side JavaScript or in an integrated fashion using server side NodeJS.

1. Image analysis is performed by Evolve2 (0.08)[22] the results of which are exported in BioNavigator’s crosstab format separately as 2 files: Median signal and Background with all possible metadata. Additionally, all images captured during the run are added to a directory on the server.
2. A data parser creates JSON documents for lvl 1.0.0 and basic names data from the crosstab format. These data are added to the database as part of the appropriate collections.
3. Outlier detection is performed on the lvl 1.0.0 data creating lvl 1.0.1 documents. This is based on temporary shifting, curve fitting to detect outliers, followed by permanent shifting of kinetic data. If multiple exposure time were utilized for kinetic curves, the slopes created by varying exposure times are added here as an additional kinetic curve, cycle series.
4. A linear regression-based background-smoothing algorithm is applied to lvl 1.0.1 to create lvl 1.1.2 data objects.
5. Final curve parameterization is performed on both lvl 1.0.1 and lvl 1.1.2 data objects. These create lvl 2.0.1 and lvl 2.1.2 parameter objects respectively.

### Outlier detection

Data points were automatically marked as potential outliers when the following was true: $e^{2}>16\sigma_{e}^{2}$ where e is an individual error residue and $\sigma_{e}^{2}$ is the variance of the error for a given model. $\sigma_{e}^{2}$ was estimated from the variance of all errors separately from both linear and non-linear models. A fit containing at least one outlier then undergoes iterative fitting, dropping each point in sequence. Based on each fit an adjusted *R*^2^ is calculated, the best *R*^2^ is removed from the list, and a mean $\overset{-}{R^{2}}$ and standard deviation, $s_{R^{2}}$ are calculated. If the best model $R^{2}>\overset{-}{R^{2}}+2s_{R^{2}}$ the corresponding point is called an outlier. This is done recursively up to 4 times or until the number of points for a linear model is < 4 or kinetic model < 9. These models used as a basis for this are described below in Methods: Data Parameterization.

### Background normalization

This procedure can be applied to each image separately. For a given image, all values are shifted by *y*_0_ so the lowest value is 0. Every point is then collected along with neighboring backgrounds into the following equation, and the ***β*** parameter matrix is solved for:

$$\left[\begin{array}{c} b_{0}\\ \vdots \\ b_{144} \end{array}\right]=\left[\begin{array}{cccc} 1 & y_{1} & b_{1,1} & b_{2,1}\\ \vdots & \vdots & \vdots & \vdots \\ 1 & y_{144} & b_{1,144} & b_{2,144} \end{array}\right]\left[\begin{array}{c} \beta_{0}\\ \beta_{1}\\ \beta_{2}\\ \beta_{3} \end{array}\right]$$ (1)

Where *b*_*i*_ is the background for spot*i*, *y*_*i*_ is the signal for spot*i*, *b*_1,*i*_ is the average background spots directly above and below spot*i*, *b*_2,*i*_ is the average background for spots directly diagonal from spot*i*. Once the ***β*** parameter matrix is solved the signal values are returned to their original value and the new background values are set as follows:

$$\hat{b_{i}}=\beta_{0}+\beta_{1}y_{1}+\beta_{2}b_{1,i}+\beta_{3}b_{2,i}$$ (2)

### Data parameterization

All data were treated separately as background and signal. This is due to the differences in analytic techniques used across the literature and due to the more robust fitting of lower signal spots when data is treated separately. All kinetic data for a given exposure time had the minimum background across all data subtracted. This sets the baseline at an acceptable place for the following model:

$$y\left(c\right)=\frac{y_{max}\cdot v_{i}\cdot \left(c-c_{0}\right)}{y_{max}+v_{i,}\cdot \left(c-c_{0}\right)}$$ (3)

Where *y*_*max*_ is the predicted maximum value for the kinetic curve, *v*_*i*_ is the initial velocity parameter; *c*_*0*_ is an adjustment parameter. The linear (post-wash) data was fit utilizing a standard linear model as were all image series taken throughout the kinetic reads.

y(e)=mx+b (4)

The complexity of this particular data set makes some additional notation helpful.

*m* Slope of the line formed by the values from the post-wash analysis.
*m*_*c*_ Slope of the line formed by the values from a given cycle number. In this experiment it takes values from 32 to 152. (cycles)
*v*_*i*,·_ Initial velocity parameter from Eq 3 utilizing all *m*_*c*_ as y values.
*v*_*i*,*e*_ Initial velocity parameter from Eq 3, for a given exposure time ‘e’. Takes values of 10, 20, 50, 100, 200 (ms).

All indicators add a subscript *b* when describing exclusively the background or $\hat{b}$ when discussing background corrected values. For the most part, we will be focusing on *v*_*i*,50_ and *m*. This is because the majority of literature utilizes these values.

When accounting for background current methods either ignore background (less common) or correct for it in the following manner:

$$\text{ls100}\left(p\left(s\right),\;p\left(b\right)\right)={\text{log}}_{2}\left(100\left(p\left(s\right)-p\left(b\right)+c_{e}\right)\right)$$ (5)

Where the corrected value is the *ls*100, *p*(*s*) is the parameter value for the signal *s* curve of interest, *p*(*b*) is the corresponding background parameter (for either b or $\hat{b}$) and *c*_*e*_ is a correction factor. *c*_*e*_ is determined by pooling all *s*−*b* across an experiment, by subtracting the 5^th^ quantile, V1, from 1: *c*_*e*_ = 1−*V*1({*s*_1_−*b*_1_,…, *s*_*n*_−*b*_*n*_}). Unfortunately, this correction factor is highly dependent on the number of samples and must be rederived for sample subsets or meta-analyses of grouped experiments. To overcome this experiment-specific correction factor, we propose an alternative approach that accounts for background by the following:

$$ls\left(p\left(s\right),\;p\left(b\right)\right)={\text{log}}_{2}\left(\frac{p\left(s\right)}{p\left(b\right)}\right)$$ (6)

### Reproducibility

#### Measurement reproducibility

In this case, we refer to measurement reproducibility as the accuracy of the values as obtained by a single image analysis step. To determine this, we utilize different camera exposure times for the parameterization of kinetic curves. This allows the parameterization of Eq 3 for a more robust signal value (*v*_*i*,·_) to be compared to that obtained in a less robust manner (*v*_*i*,*e*_). For this comparison, we treat the 12 samples independently with a correlation being calculated based on one (*v*_*i*,*e*_) to one (*v*_*i*,·_).

#### Technical reproducibility

Technical replicates are the same sample run on multiple PamChip arrays. For this, we have 4 samples with 3 technical replicates each[29]. Correlations were based on 1–2 comparison where replicates are paired in all possible ways transforming the 3 measurements to 6 (x, y) points ({*x*,*y*,*z*} → {(*x*,*y*), (*x*,*z*), (*y*,*x*), (*y*,*z*), (*z*,*x*), (*z*,*y*)}. To determine if the reproducibility is a factor of sample or random, 4 random groups of 3 were formed 10 times over and the same correlations were calculated.

### Database: Application program interface

We utilized JavaScript Object Notation (JSON) Objects for data representation. This is due to its relative ubiquity as parsable data and its ability to represent the complex structure without significant repetition. Data models for the 3 major levels can be seen in S3–S5 Figs. In addition to the 3 major levels, the minor levels are described in S1 Table and S2 Fig.

MongoDB 3.4 was utilized as the database backend. A small read-only server was created with NodeJS v 8.0.0 (NodeJS) with the packages: restifyJS v4.3.0, and MongoDB 2.2.6. This server is utilized to serve MongoDB documents and captured images. The documents are available at http://db.kinomecore.com/db/1.0.0/{level}/<params>. The images are available at: http://db.kinomecore.com/image/{img_name}. Full API documentation with swagger can be found https://app.swaggerhub.com/apis/adussaq/KINOME/1.0.0. The code for creating your own backend server along with full documentation of its use can be found https://github.com/kinome/kinome_toolbox. The MongoDB server is housed at UAB as part of our research cluster. The restifyJS server code is viewable here: https://github.com/kinome/kinome_toolbox/blob/master/server.js.

## Results

### Kinome toolbox development

**Fig 1:**
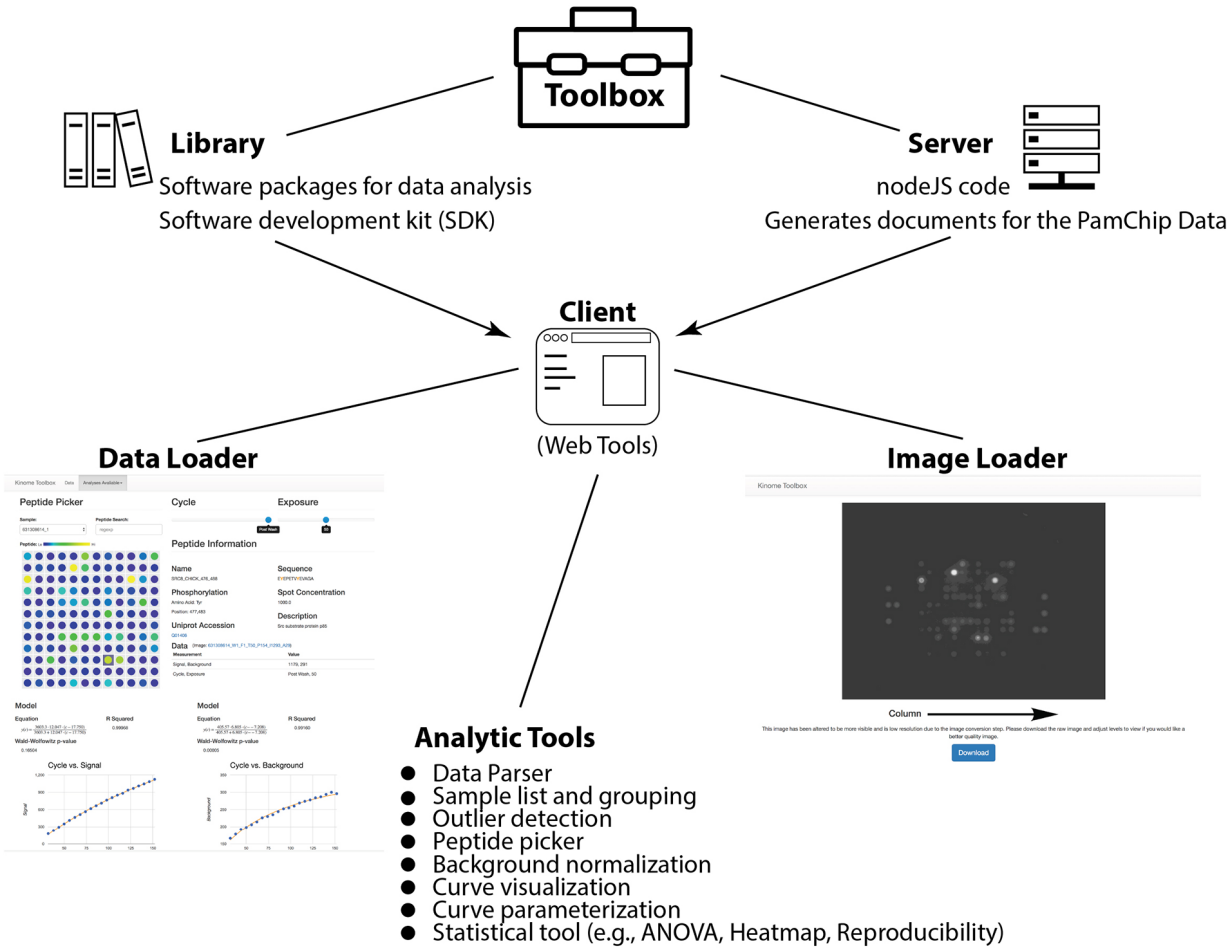
Kinome toolbox schematic. A schematic of the toolbox organization and analytic tools are shown. The three major components are the Library, Server, and Client.

The kinome toolbox is a series of tools utilized for the processing and visualization of PamChip kinomic peptide arrays. The client toolbox is available at http://toolbox.kinomecore.com/ and the code for all three parts is available at https://github.com/kinome/kinome_toolbox. It is written in JavaScript for both the client and the server and has three major components as described below (Schematically shown in Fig 1).

Library. The library is a series of packages that can be utilized to convert data between levels and to enrich the kinome objects with a rich software development kit (SDK) for easier use. This SDK is documented https://github.com/kinome/kinome_toolbox.

Server. The server component exists to quickly create the documents for all major and minor levels from the input of a Bionavigator crosstab file. It is written in nodeJS and uses a flag-based command line interface.

Client. The client consists of a series of tools designed to make working with kinome data as simple as possible. It has two major pages: (1) an image loader that accepts an image name and displays the array image associated and (2) a data loader that creates a contextually determined home page.

The image loader utilizes the Kinome API to pull the original run images from the server. It converts the tiff to the standard RBG color scheme and utilizes those values to brighten the image to make them more visible. Additionally, a link to download the original high-quality tiff is provided.

The home page loads in data, code, and styles by URL parameters. The data loading blocks code loading and styles load asynchronously. Data is cached for 30 minutes by default, and up to 90 days if it is determined to be unique mongo documents. Styles are cached for 90 days. These in combination decrease revisit load time significantly. Additionally, once data is loaded the page determines the data type present and displays a series of available functions. The entire library can be explored at https://github.com/kinome/kinome_toolbox. Additionally, in order to more fully explain the toolbox, there is a YouTube playlist that describes the basic use of the tool available here: http://bit.kinomecore.com/?playlist. The kinomics toolbox will by default display our public names database and a table to build comparison groups. To build up the other pages or add samples to analytic groups, follow the links that appear below the table. Or you can go here: http://bit.kinomecore.com/?p1_1.0.0 to see all the level 1.0.0 data used in this manuscript and be provided links to visualize all other data levels.

At the time of writing this, the visualizations and analyses available for level one were: outlier detection, background normalization, and general visualization. For level two: reproducibility (by groups), measurement reproducibility (by sample), ANOVA (by groups), and Heatmap (by samples, displays group identity). Level one data provides links to view and download the original images for points of interest. Level 1 visualization displays a virtual array, a sample, cycle and exposure time selector. The virtual array is colored by signal—sample to give relative signal strength. Clicking on a peptide in the virtual array will pull up specific values for that peptide, display the peptide meta data, and provide a link to the image associated with the selected cycle/exposure combination. This visualization allows data inspection at all levels and in combination with the meta data displayed in the main table, provides the meta data needed to meet the majority of MIAME criteria.

### Case study

The protein MARCKS has been implicated in GBM biology by our lab and others including effects on proliferation, invasion, DNA damage repair, nuclear localization and signal transduction cascades. The MARCKS effector domain (ED) is a critical region of the protein allowing it to reversibly bind to phosphatidyl inositol (3,4) bisphosphate (PIP2), calcium-calmodulin (CaM), and actin while also serving as its nuclear localization signal. We generated doxycycline-inducible MARCKS ED mutants in the U87 GBM cell line as described in Methods and S1 Fig. The four mutants were induced to overexpress and were kinomically profiled. Using these isogenic lines, we will demonstrate the kinome toolbox functionality in the subsequent figures. We will show replicate grouping assignment, outlier detection, background normalization, curve fitting with data parameterization, statistical testing with simple ANOVA and hierarchical clustering for heatmap visualization. Video tutorials and URL’s for individual figures are provided in the appropriate sections.

#### Data grouping and outlier detection

**Fig 2:**
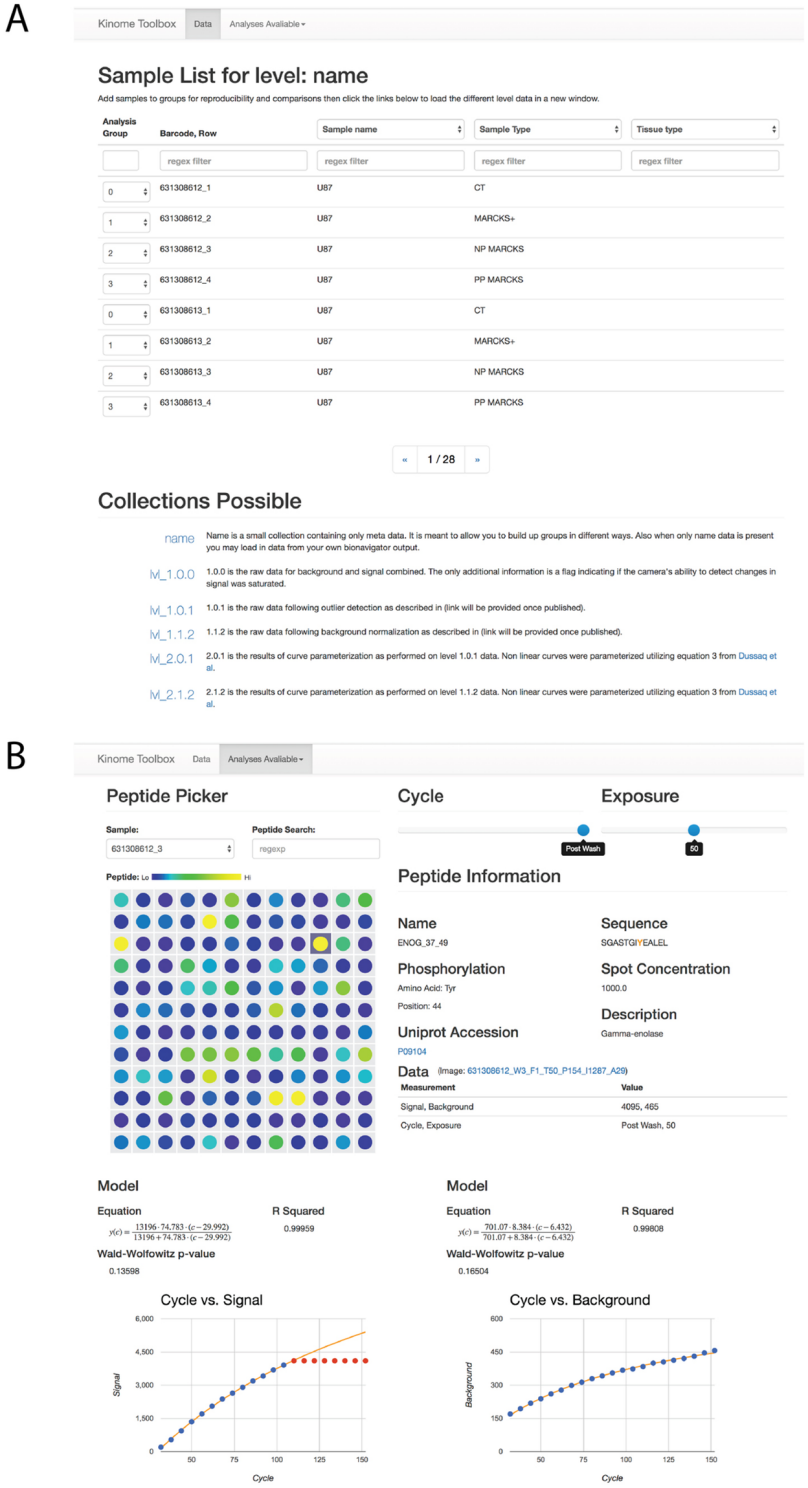
Data grouping and outlier detection. A) shows the sample list screen under the Data tab. Drop down menu allows for selecting samples to be analyzed as particular groups. The data levels can be selected using the links at the bottom with corresponding descriptions. B) shows the Data Visualization tool under the Analyses Available tab. The peptide ENOG_37_49 has been selected and peptide information, cycle number, exposure, data measurements and curve fits are shown. Red dots on the fitted curve indicate spots that were auto-flagged as outliers using the stringent cutoffs described in the Results section.

Each replicate of the four mutant lines were assigned to Analysis Groups (Control or CT = Group 0; MARCKS wild-type overexpression or MARCKS+ = Group 1; non-phosphorylatable MARCKS or NP MARCKS = Group 2; and pseudophosphorylatable MARCKS or PP MARCKS = Group 3) under the data tab. Outliers were then removed from the data using the Analysis tab as described in Methods. For linear models $16\sigma_{e}^{2}=56.9441$, 0.81% (3,092/ 378,970) of all data points exceed this error threshold and 2.23% of fits (1,694 of 76,032). For non-linear models $16\sigma_{e}^{2}=262.3680$, this represents 0.85% (3,060/361,798) of all data points and 4.17% of fits (721/17,280). Once filtered, this lead to 0.74% (3,393/455,002, including *m*_*c*_) of points being determined to be outliers. An example of an auto-flagged outlier for the ENOG_37_49 probe on Sample 631308612_3 is shown as red dots on the Cycle v. Signal curve within the Data Visualization tool (Fig 2) where the signal intensity exceeds the linear range of the camera. Removal of the outliers provides a well fit curve (R^2^ = 0.99959). We believe these cutoffs are a reasonable starting point for future data analysis.

#### Background normalization

**Fig 3:**
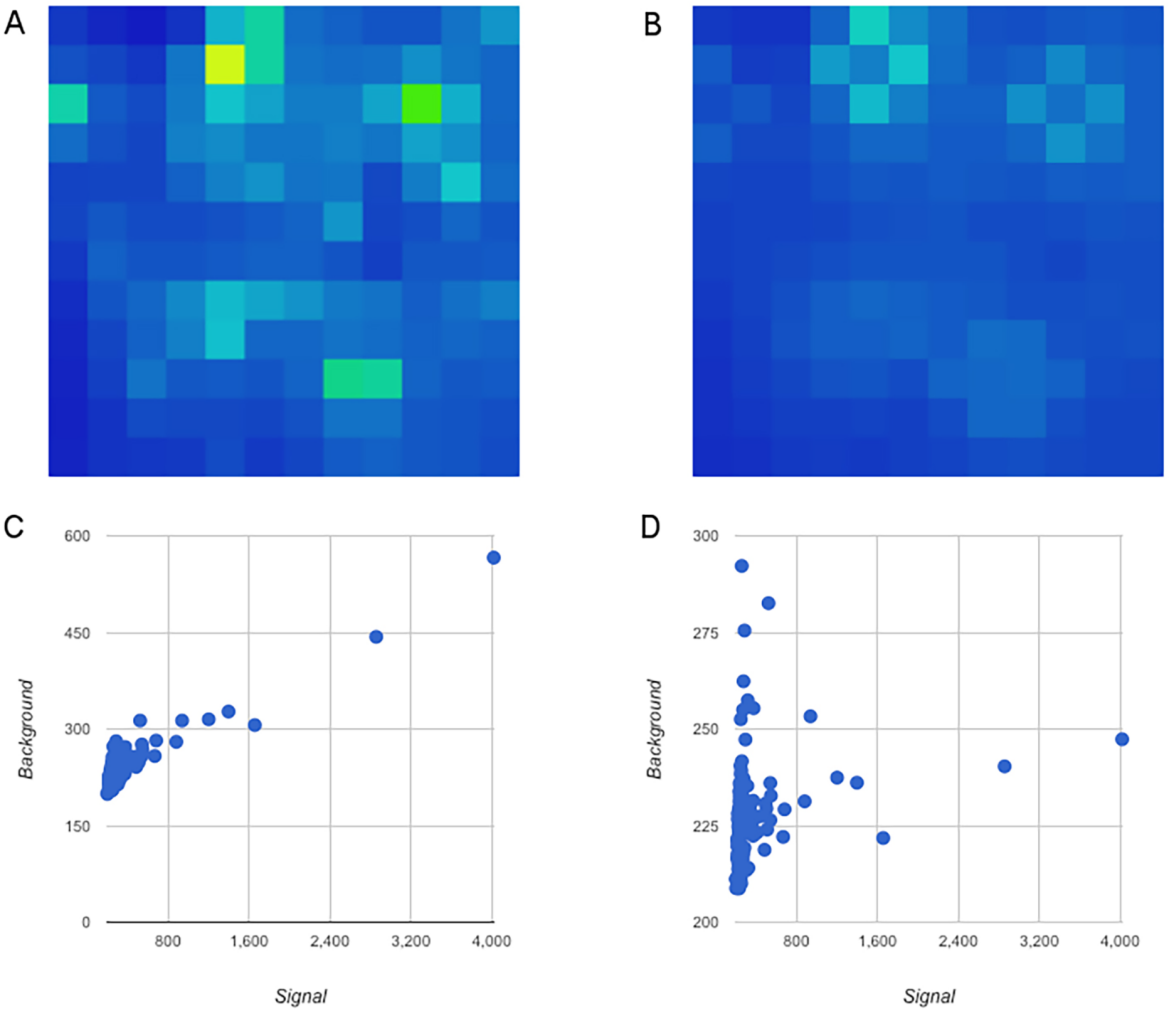
Background correction of a single array. One array image was chosen for visualization. Panel A and C represent the background before normalization. Panel A shows the background values plotted against position. Panel C shows the spot intensity versus background intensity and has a correlation of 0.9642 (p-val: <0.0001). Panels B and D represent the background after normalization. Panel B is background plotted against position. Panel D is spot intensity versus background intensity and has a correlation of 0.2272 (p-val: 0.0062). These figures can be generated for every image presented here at http://bit.kinomecore.com/?fig3.

As mentioned earlier, publications using this platform have typically utilized a median signal minus immediate background for calculating peptide intensities. However, since our kinome toolbox handles the signal and background intensities separately, we noted that the background itself is a function of the corresponding signal—in other words, adjacent higher intensity spots will influence the background intensity. This creates a situation where subtracting the background without normalization dampens the signal. Fig 3 depicts a single image before and after background correction. It can be seen that the correlation between background and signal nearly disappears as a result of the correction. This also has the effect of decreasing the background variance across the chip as can be seen in Fig 3 panel B. We will assess the differences between these approaches in the Reproducibility section below.

#### Data parameterization

Treating background and signal separately lead to very successful fitting. Linear (Post Wash) signals averaged an R^2^ of 0.9985 ± 0.030 and kinetic (Cycle) signals averaged an R^2^ of 0.9903 ± 0.012. Background signals averaged an R^2^ of 0.9985 for linear fits and 0.9833 for kinetic fits. Corrected background ($\hat{b}$) signals averaged an R^2^ of 0.9988 for linear fits and 0.9922 for kinetic fits. *c*_*e*_ was calculated for all parameters of potential interest, all these values are displayed in the client side tool, key ones include: 1.0610 for ls100(*m*(*s*), *m*(*b*)), 1.0047 for $\text{ls100}(m(s),\;m(\hat{b}))$, 1.0478 for ls100(*v*_*i*,·_(*s*), *v*_*i*,·_(*b*)), and 1.0019 for $\text{ls100}(v_{i,\cdot }(s),\;v_{i,\cdot }(\hat{b}))$. We will assess the differences between these approaches in the Reproducibility and Clustering sections.

#### Reproducibility

One of the challenges in the field of kinomic peptide arrays relates to accuracy determination across arrays because there is no accepted “background” kinase activity for comparison as opposed to the transcriptomic "housekeeping gene" that is traditionally used for array normalization. As such, we use technical reproducibility as a proxy to determine the accuracy of the data and data manipulations. Here we propose an additional, more fundamental measurement, *v*_*i*,·_ as the standard to compare individual *v*_*i*,*e*_ to assess measurement accuracy.

#### Measurement reproducibility

We will look at measurement reproducibility with two goals in mind. (1): Determine the added value of capturing multiple images (by varying camera exposure time) rather than the standard single exposure (50 msec) during the kinetic phase. (2): Determine the effectiveness of background correction in producing consistent (i.e, improved Spearman correlations) results across image series.

**Fig 4:**
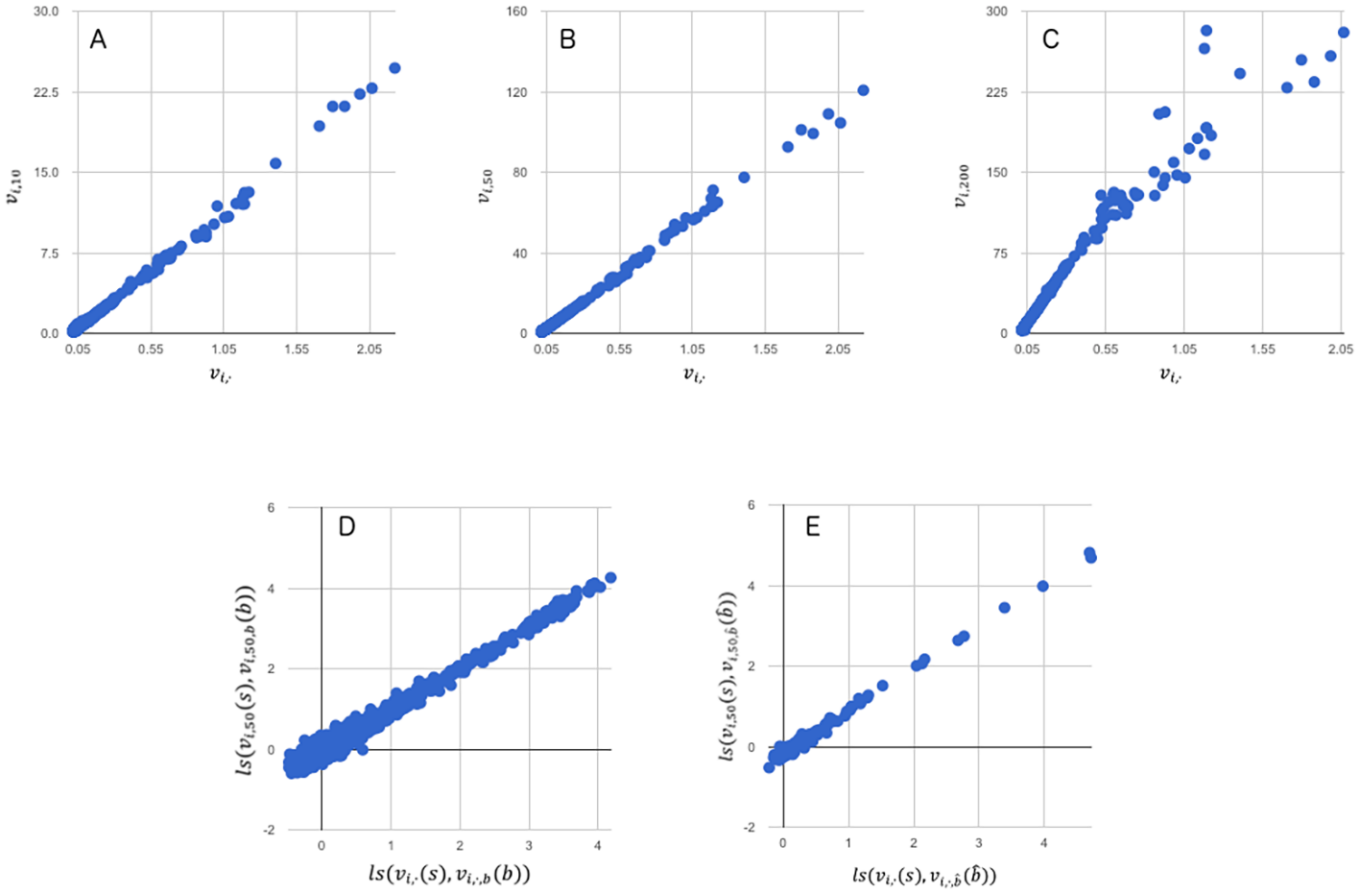
Measurement reproducibility for kinetic parameterization. In panels A-C, kinetic curves created by images series of varying exposure times (*v*_*i*,e_) are compared to the more robust *v*_*i*,·_. The data presented is 144 peptides for all 12 samples discussed in Methods. Panel A is a 10 ms exposure time and has a Pearson's r = 0.9981 and a Spearman's ρ = 0.9097. Panel B is a 50 ms exposure time and has a Pearson's r = 0.9989 and a Spearman's ρ = 0.9920. Panel C is a 200 ms exposure time and has a Pearson's r = 0.9807 and a Spearman's ρ = 0.9995. Panels D compares *ls*(*v*_*i*,50_ (*s*), *v*_*i*,50,*b*_(*b*)) to *ls*(*v*_*i*,·_ (*s*), *v*_*i*,·,*b*_(*b*)) and has a Pearson's r = 0.9887 and a Spearman's ρ = 0.9279. Panels E compares $ls(v_{i,50}(s),\;v_{i,50,\hat{b}}(\hat{b}))$ to $ls(v_{i,\cdot }(s),\;v_{i,\cdot ,\hat{b}}(\hat{b}))$ and has a Pearson's r = 0.9941; Spearman's ρ = 0.9681. These figures and all similar variations can be further investigated and explored at http://bit.kinomecore.com/?fig4a for *b* and at http://bit.kinomecore.com/?fig4b for $\hat{b}$.

1. The correlation of the kinetic parameters *v*_*i*,*e*_ for various exposure times, *e*, to the more robust *v*_*i*,·_ is shown in Fig 4. In the shorter exposure times, the noise introduced is in the low signal tail, while the longer exposure times have noise in the higher signal range. For example, when comparing the *v*_*i*,10_ for all samples to *v*_*i*,·_ we get a Pearson’s r = 0.9981 and Spearman's ρ = 0.9097. With the lower rank correlation being caused by low resolution of the lower signals. However, for *v*_*i*,200_ versus *v*_*i*,·_ we see the opposite with Pearson's r = 0.9807 and Spearman's ρ = 0.9995. The rank correlation increases to further separate signals, however, the high data points become more erratic. Taken together this indicates that the cycle slope (*v*_*i*,·_) may provide additional information not fully captured with a single exposure time.
2. To determine how background normalization affects the reproducibility of measurements at the standard 50ms exposure time for kinetic studies, we want to compare the correlation of *ls*(*v*_*i*,50_ (*s*), *v*_*i*,50,*b*_(*b*)) and *ls*(*v*_*i*,·_ (*s*), *v*_*i*,·,*b*_(*b*)) to the correlation of $ls(v_{i,50}(s),\;v_{i,50,\hat{b}}(\hat{b}))$ to $ls(v_{i,\cdot }(s),\;v_{i,\cdot ,\hat{b}}(\hat{b}))$. We can see the results of this in Fig 4 where both measures of correlation improve when utilizing the background normalization. The change in Pearson’s R represents a significant difference (p-val: < 0.0001 Fisher). Interestingly the difference increases for lower exposure times with the $\hat{b}$ outperforming *b*, however for higher exposure times this pattern switches, favoring the uncorrected *b*. Taken together these observations suggest multiple time points and background correction may help distinguish lower signal spots.

#### Technical reproducibility

We further addressed the utility of background normalization and multiple camera exposures in the kinetic studies by focusing on 3 questions related to technical reproducibility. (1) Does background normalization improve the reproducibility of linear, post wash slope, *m*, or the kinetic initial velocity parameter, *v*_*i*,50_? (2) Does using ls(·) to account for the background improve the reproducibility of kinetic or post wash key parameters (*m*,*v*_*i*,50_) over ls100(·)? (3) Does *v*_*i*,·_ improve the reproducibility of kinetic parameterization over *v*_*i*,50_? The correlations used to discuss these questions are shown in Table 1.

**Table 1:** Reproducibility of background corrected technical replicates. The correlation of the parameters for 144 peptides across 4 sets of 3 technical replicates (3,456 points). These and similar comparisons can be visualized for background normalized data: http://bit.kinomecore.com/?tab1a and for non-normalized: http://bit.kinomecore.com/?tab1b.

Equation	Signal Parameters	Spearman Correlation	Equation	Signal Parameters	Spearman Correlation
ls100(·)	*m*,*b*	0.99075*	ls(·)	*m*,*b*	0.99018
ls100(·)	*m*,$\hat{b}$	0.98768	ls(·)	*m*,$\hat{b}$	0.99240*
ls100(·)	*v*_*i*,50_ *b*	0.97973	ls(·)	*v*_*i*,50_, *b*	0.96991
ls100(·)	*v*_*i*,50_, $\hat{b}$	0.98192	ls(·)	*v*_*i*,50_, $\hat{b}$	0.98246
ls100(·)	*v*_*i*,·_,*b*	0.97477	ls(·)	*v*_*i*,·_,*b*	0.97817 **
ls100(·)	*v*_*i*,·_, $\hat{b}$	0.97430	ls(·)	*v*_*i*,·_, $\hat{b}$	0.98334 **

*, ** p-val < 0.0001 by 2 tail Fisher approximation.

All four investigation groups show similar results for questions (1) and (2). The background corrected ls(·) has the highest correlation throughout, significantly outperforming the next highest for both *v*_*i*,·_, and *m*, but not for *v*_*i*,50_ (p-val: 0.6965, 2 tail Fisher approximation). While this is reasonably clear, the pairwise comparisons are murkier. Depending on the equation group the older methodologies actually will outperform the newer ones when comparing ls100(·, *b*) to $\text{ls100}(∙,\hat{b})$, for instance, both *m* and *v*_*i*,·_ have higher values without background correction (p-val: <0.0001 and 0.7872 respectively). Similarly, the older method of ls100(·, *b*) outperforms the newer ls(·, *b*) in two cases. This indicates that the combination of cycle slope with background correction provides a slight improvement in correlations over standard methods while incorporating only one of the new parameters (cycle slope or background correction) had minimal impact.

(2) Based on these data alone, the added value of the multiple kinetic exposure images is questionable. Two of the four pairwise comparisons favor using *v*_*i*,50_, (ls100(·, *b*), $\text{ls}100(∙,\hat{b})$ p-vals <0.001), and two favor using *v*_*i*,·_ (ls(·, *b*), $\text{ls}(∙,\hat{b})$ p-val < 0.0001, p-val = 0.5). As such, the benefit of multiple kinetic exposures may be limited to select experiments such as those with a) high signal spots that will remain in the linear range of the camera for a larger portion of the kinetic phase of the assay or b) low signal spots that may have modestly enhanced resolution.

Because the cell lines utilized in this case, study are isogenic, we expect a fairly large amount of kinomic overlap. As such, we repeated the reproducibility calculations using 10 rounds of random sampling to create 4 groups of three ‘pseudo-technical replicates’ to generate a baseline level of reproducibility. For $\text{ls}(m,\hat{b})$ we have 0.968±0.007, for $\text{ls}(v_{i,50},\hat{b})$ we have 0.956±0.007 and for $\text{ls}(v_{i,\cdot },\hat{b})$ we have 0.949±0.010. These values all indicate that the technical reproducibility is indeed higher for actual technical replicates than it is for random sample groups. Additionally, the fact that the baseline level reproducibility for $\text{ls}(v_{i,\cdot },\hat{b})$ is lower than that of $\text{ls}(v_{i,50},\hat{b})$ suggests we may be getting benefits from the multiple exposure times not immediately obvious from Table 1.

#### Differential display tools (ANOVA and hierarchical clustering heatmap)

In addition to the tools described above, that focused on determining data quality we created two tools for visualizing differences between sample groups, an ANOVA display, and a Heatmap with hierarchal clustering to find the most related samples. Based on the results above, we will focus on $\text{ls}(m,\hat{b})$ and $\text{ls}(v_{i,\cdot },\hat{b})$. We begin by applying one-way ANOVAs to each peptide separately for $\text{ls}(m,\hat{b})$ then for $\text{ls}(v_{i,\cdot },\hat{b})$. Table 2 lists the 5 most category deterministic peptides found in this manner for each dataset. These are expressed and ranked by the F-statistic and not p-value due to the fact that the reporting of a p-value would inflate the interpretation of these differences, as they do not account for a number of prior distributions or multiple testing errors. It is interesting to note that despite the relationship between the measurements, the kinetic and end level data provide a slightly different top 5 peptides.

**Table 2:** List of the top 5 differentially phosphorylated peptides as determined by one-way ANOVA f-statistic. Ranks are indicated in parenthesis. These values and many more can be visually inspected at http://bit.kinomecore.com/?tab2.

Peptide	F-statistic (rank), $\text{ls}(m,\hat{b})$	F-statistic (rank), $\text{ls}(v_{i,\cdot },\hat{b})$	Sequence	Source Uniprot ID
src8_chick_492_504	44.3447 (7)	83.0656 (1)	YQAEENTYDEYEN	Q01406
efs_246_258	77.6595 (1)	22.7558 (8)	GGTDEGIYDVPLL	O43281
paxi_24_36	61.6226 (4)	20.8223 (10)	FLSEETPYSYPTG	P49023
pgfrb_572_584	73.8763 (2)	36.1151 (3)	VSSDGHEYIYVDP	P09619
enog_37_49	49.8139 (5)	25.9734 (6)	SGASTGIYEALEL	P09104
frk_380_392	67.0898 (3)	42.2960 (2)	KVDNEDIYESRHE	P42685
pgfrb_1002_1014	21.7951 (18)	34.7926 (4)	LDTSSVLYTAVQP	P09619
cdk2_8_20	12.0567 (29)	28.3214 (5)	EKIGEGTYGVVYK	P24941

**Fig 5:**
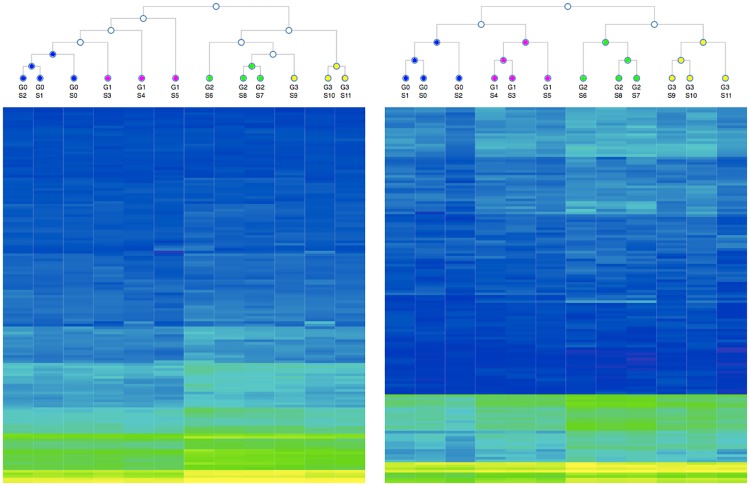
2-way hierarchal clustering of technical replicates as visualized by heatmap. Panel A is based on $\text{ls}(m,\hat{b})$ and panel B is based on $\text{ls}(v_{i,\cdot },\hat{b})$. The cluster diagram does not represent the distance between branches, only the branch points of a hierarchal clustering model based on Euclidean distance with average-linkage clustering. Yellow is the highest signal peptides, blue is the lowest. The peptide order varies between the two panels and are based on an identical hierarchal clustering. These figures and several more can be inspected at http://bit.kinomecore.com/?fig5.

One of the most common ways to visualize and utilize microarray data, such as PamChip data, is as a heatmap with a clustering algorithm. In general, this is performed to identify groupings that may share characteristics such as a molecular subtype or susceptibility to a particular kinase inhibitor. Fig 5 is a heatmap and two-way hierarchical cluster (using Euclidian distances and average-linkage clustering) for both $\text{ls}(m,\hat{b})$ and $\text{ls}(v_{i,\cdot },\hat{b})$ separately. Despite the isogenicity of the 12 samples, clustering based off both curve fitting equations separate the groups well with $\text{ls}(v_{i,\cdot },\hat{b})$ actually creating “perfect” clustering (i.e., each technical replicate for a given analysis group clustered closest to the other replicates in the same group). While other parameter and equation groupings produce similar results, no other parameter combination tested reproduces this “perfect” clustering. This separation lends further credence to the potential utility of *v*_*i*,·_ in future experiments.

### Recommendations

#### Kinetic curve capture

The multiple image capture does not significantly change the reproducibility of data, however, it does help clarify low signal spot and allows high signal spots to remain measurable for longer. Additionally, the clustering for the technical replicates based on the $\text{ls}(v_{i,\cdot },\hat{b})$ outperforms all other clustering methods. While this is a small sample set, there does seem to be potential in using multiple exposure times, and we believe the results here justify further investigation and use.

#### Background normalization and correction

The new techniques presented here clearly increased the reproducibility of the data and provided better clarity data. Going forward it is clear that an additional level of data is needed to further simplify the downstream analysis. Based on the technical reproducibility results, and the lack of an experiment-specific correction factor (***c***_***e***_), we recommend utilizing both background correction in conjunction with ***ls***(**·**).

## Discussion

Directly investigating the kinome gives us a fundamental picture of cellular regulation that would be hard, if not impossible, to access from solely genomic and transcriptomic information. Kinome arrays such as the PamChip offer a unique look at cellular kinase activity in the form of measuring the activity of entire kinomes on a series of known kinase targets. Where other kinome arrays offer only end level data, the PamChip provides a measure for kinetic data in the form of segmented image capture over the course of a reaction. This allows changes in the rate of phosphorylation to be investigated and is most commonly used to compare early reaction rates in the presence of *ex vivo* kinase inhibitors[11,17].

The field of kinomics is still relatively nascent, and modern reviews still discuss the need for standardization of data formats and analytical techniques [9,23]. To this end we present a number of new ideas and methods for the PamChip microarray:

1. A standardized data format
  Our JSON format is extensible enough to fit MIAME recommendations[24] while working to address the self-critique[25] of MIAME. JSON parsers exist for numerous programming languages and JSON objects provide the framework for all web programming.
2. A pre-processing pathway
  Prior to this work, investigators directly fit either median signal or median signal-background for parameterization, we propose treating these separately. Additionally, we include an automated method for the removal of outliers and a proposed background normalization technique. Our work shows a benefit to these additional steps.
3. A public data repository and tools to create your own
  A MongoDB database is the basis of our public data repository. The server that powers it is a minimal Cross Origin Resource Sharing (CORS) enabled JSON document server. We have included the data produced for this study and intend to add more data in the future. We provide the server code and a series of tools to populate the database. With a reasonable knowledge of network management, this can be set up in just a few steps and can be set up at any institution.
4. An extensible visualization toolbox for multilevel array data.
  A major limitation for PamChip data analysis is the complexity of this data structure. We provide tools for visualization of various data levels. Additionally, by allowing developers to access a series of basic functions for interacting with data we have created a system that can greatly ease software development. This creates an ecosystem for web programmers to quickly begin an analysis with minimal knowledge of the data structure.

As discussed in methods, the kinomics toolbox provides an SDK for interacting programmatically with the data. An author on this paper created several of the tools described here with no prior knowledge of the data, only access to the provided SDK and a few conversations about how the data works. This was performed without any knowledge of the data model aside from meta data storage. We believe this process of tool development led to a more useful SKD and indicates the development potential. Going forward we will increase the default packages available to provide more thorough and robust data analyses.

## Supporting information

S1 Fig Schematic of mutations made to MARCKS Non-Phospho (NP) and MARCKS Pseudo-Phospho (PP) proteins. (PNG)

S2 Fig Flowchart of analytical steps. This compares the analytical steps proposed here to previous analytic steps. Additionally, this describes the relationship of the different data levels to one another.
(PNG)

S3 Fig Schematic of names documents as stored in MongoDB and as utilized by the kinomics toolbox. For more information visit the full documentation site for the database: https://app.swaggerhub.com/apis/adussaq/KINOME/1.0.0.
(PNG)

S4 Fig Schematic of level 1 data documents as stored in MongoDB and as utilized by the kinomics toolbox. For more information visit the full documentation site for the database: https://app.swaggerhub.com/apis/adussaq/KINOME/1.0.0.
(PNG)

S5 Fig Schematic of level 2 data documents as stored in MongoDB and as utilized by the kinomics toolbox. For more information visit the full documentation site for the database: https://app.swaggerhub.com/apis/adussaq/KINOME/1.0.0.
(PNG)

S1 Table List of major data levels utilized in the implementation of the code and databases. (PDF)

S1 Dataset JSON Lvl 1.0.0 case study data. The level 1.0.0 dataset is available as JSON object.
(JSON)
