# Supplementary figures and images for: Kinomics toolbox—A web platform for analysis and viewing of kinomic peptide array data
Source: PLoS One. 2018 Aug 21;13(8):e0202139. doi: 10.1371/journal.pone.0202139 (PMC6103510; doi:10.1371/journal.pone.0202139)

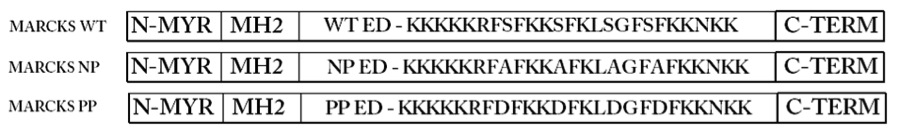

Supplement: S1 Fig — (PNG) [file pone.0202139.s001.png]

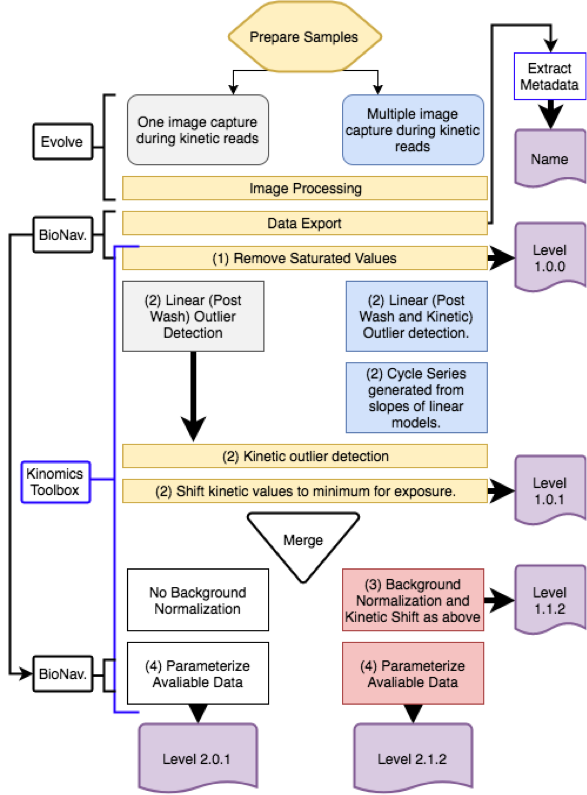

Supplement: S2 Fig — This compares the analytical steps proposed here to previous analytic steps. Additionally, this describes the relationship of the different data levels to one another. (PNG) [file pone.0202139.s002.png]

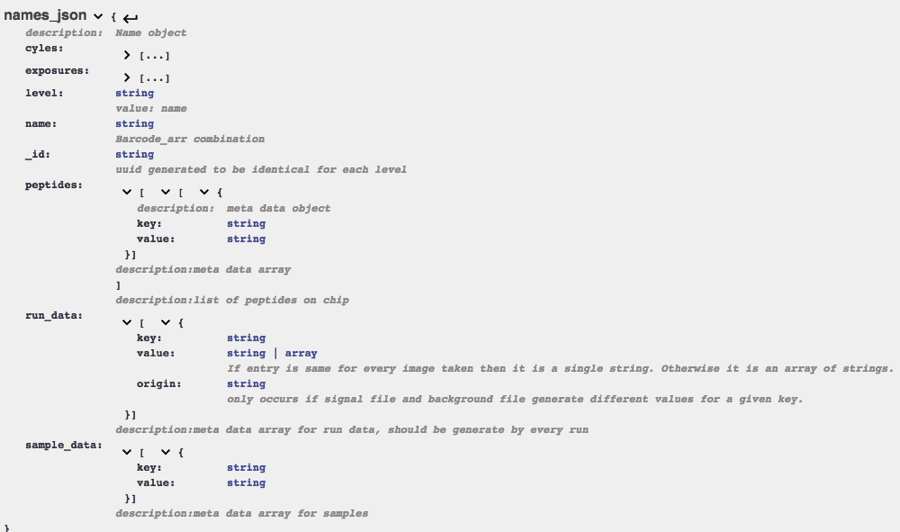

Supplement: S3 Fig — For more information visit the full documentation site for the database: https://app.swaggerhub.com/apis/adussaq/KINOME/1.0.0. (PNG) [file pone.0202139.s003.png]

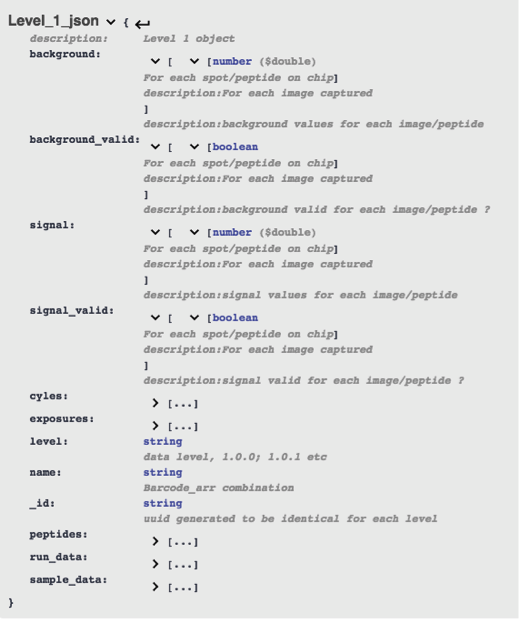

Supplement: S4 Fig — For more information visit the full documentation site for the database: https://app.swaggerhub.com/apis/adussaq/KINOME/1.0.0. (PNG) [file pone.0202139.s004.png]

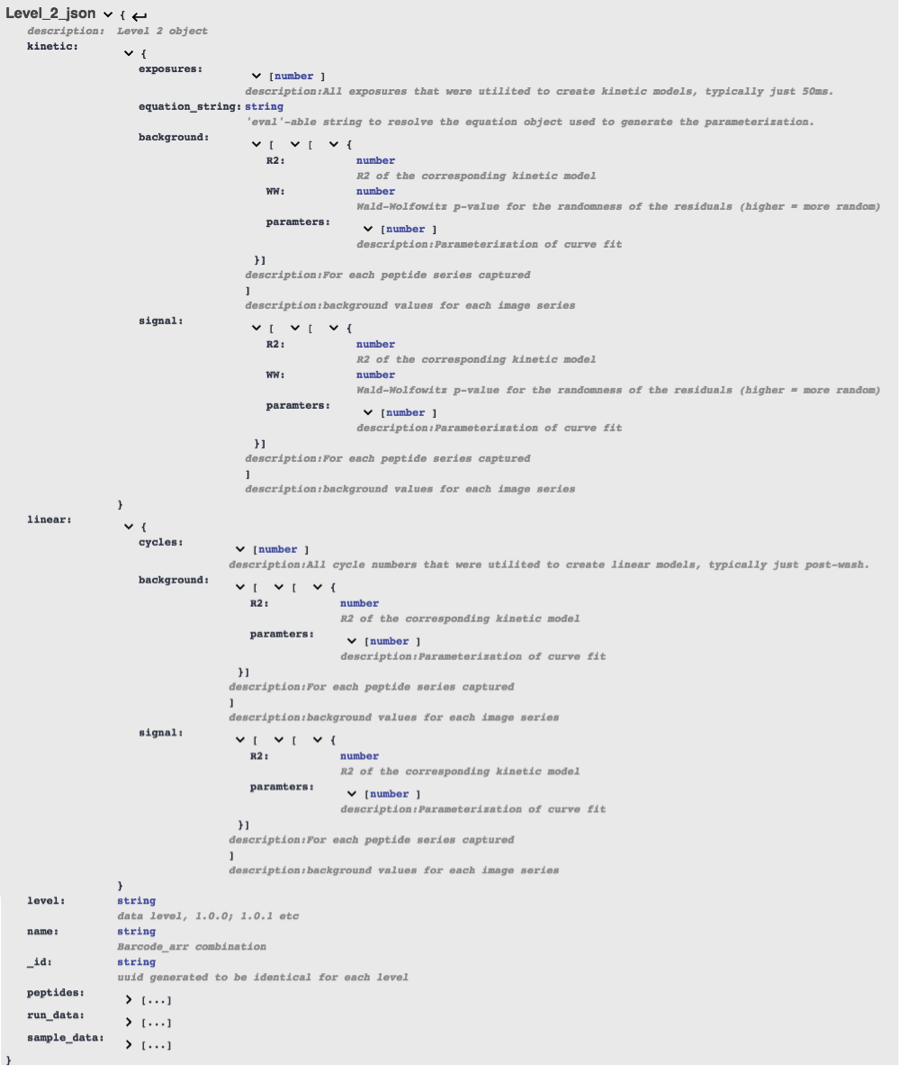

Supplement: S5 Fig — For more information visit the full documentation site for the database: https://app.swaggerhub.com/apis/adussaq/KINOME/1.0.0. (PNG) [file pone.0202139.s005.png]
