# Supplementary material for: Kinomics toolbox—A web platform for analysis and viewing of kinomic peptide array data
Source: PLoS One. 2018 Aug 21;13(8):e0202139. doi: 10.1371/journal.pone.0202139 (PMC6103510; doi:10.1371/journal.pone.0202139)
Supplement: S1 Table — (PDF) [file pone.0202139.s006.pdf]

Level	Origin	Description
names	Crosstab, Evolve2 (0.08)	This collects all the metadata to be utilized and provides the unique id that links all future data together.
1.0.0	Crosstab, Evolve2 (0.08)	Original data with added parameters for excluding data based on camera saturation.
1.0.1	1.0.0	Kinetic data shifted: $(x, y) \to (x, y - y_{0,min})$ where $y_{0,min}$ is the minimum signal or background across the initial image at the initial time point. Initial removal of outliers based on high error measures. This can be manually assisted.
1.1.2	1.0.1	Background normalized data. Our approach is described in results.
2.0.1	1.0.1	All possible linear models fit, all kinetic data fit, based on non-normalized backgrounds.
2.1.2	1.1.2	All possible linear models fit, all kinetic data fit, based on normalized backgrounds.
